# Isolated Gastric Perforation Following Blunt Abdominal Trauma: A Report of a Rare Case

**DOI:** 10.7759/cureus.91995

**Published:** 2025-09-10

**Authors:** Kradi Yassin, Soufiane Taibi, Abdelali Guellil, Haitam Soussan, Mohammed Bouziane

**Affiliations:** 1 General Surgery, Mohamed VI University Hospital, Faculty of Medicine and Pharmacy, Laboratory of Anatomy, Microsurgery and Surgery Experimental and Medical Simulation, Oujda, MAR; 2 Visceral Surgery and Digestive Oncology A, Mohammed VI University Hospital, Oujda, MAR; 3 General Surgery A, Centre Hospitalier Universitaire Mohammed VI, Oujda, MAR

**Keywords:** abdominal blunt trauma, accidents, gastric perforation, gastrique suture, pneumoperitoneumtraffic, road

## Abstract

Blunt abdominal trauma is a common result of road traffic accidents and may lead to internal injuries involving both solid organs and hollow viscera. Gastric perforations following such trauma are rare and can present significant diagnostic and management challenges. This report describes a 19-year-old male who sustained a gastric perforation after a motorcycle collision. An exploratory laparotomy revealed the perforation, which was surgically repaired with primary suturing and an omental patch. The patient recovered well postoperatively, with no complications, and was discharged in stable condition.

## Introduction

Blunt abdominal trauma is a common reason for emergency department admissions, with road traffic accidents being the most prominent cause. The spleen, kidneys, and liver are the organs most commonly injured, while gastrointestinal tract injuries are relatively rare. Gastric perforation is typically associated with penetrating trauma and is infrequently observed in blunt abdominal trauma, with a reported prevalence of 0.4% to 1.7% of all blunt abdominal injuries [1].

The diagnosis of post-traumatic gastric perforation is based on clinical examination, and a CT scan is considered the gold standard. However, a normal CT scan does not rule out the diagnosis if the clinical context is clear.

Surgical intervention is required promptly once a diagnosis of gastric perforation is established. This usually involves an exploratory laparotomy for damage repair, with a two-layer suture often being the preferred approach.

## Case presentation

A 19-year-old male with no notable medical history presented to the emergency department six hours after a road traffic accident involving a collision between a motorcycle and a vehicle, resulting in blunt abdominal trauma with an epigastric impact.

**Figure 1:**
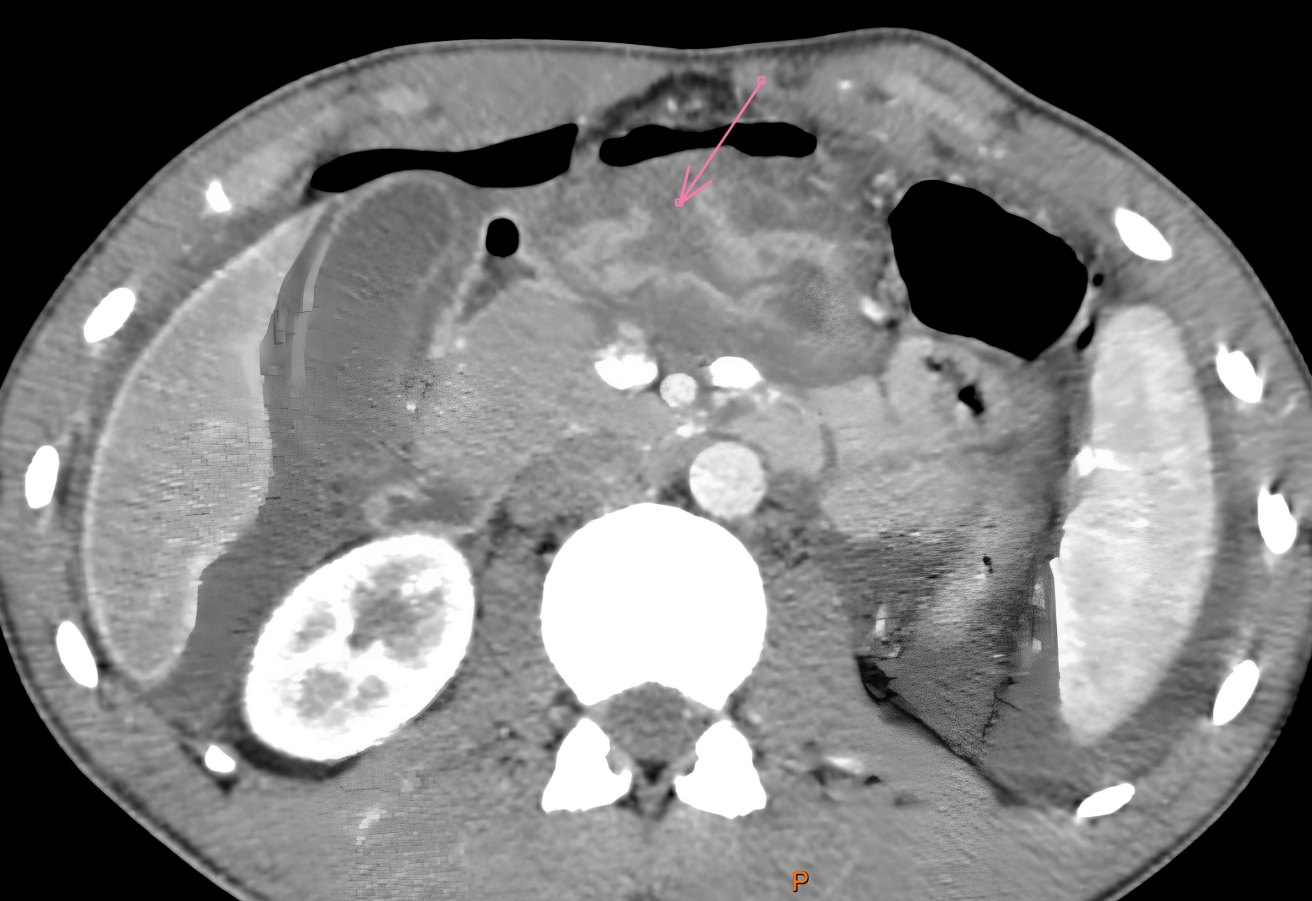
Axial computed tomography slice revealing an anterior gastric perforation

Upon admission, the patient complained of severe, diffuse abdominal pain. On examination, he was conscious, with stable hemodynamic and respiratory status. Abdominal palpation revealed diffuse tenderness and rigidity. A body scan showed significant intra-abdominal fluid, extensive pneumoperitoneum in the supramesocolic space, and a 4 cm defect in the anterior wall of the gastric antrum (Figure 1).

**Figure 2:**
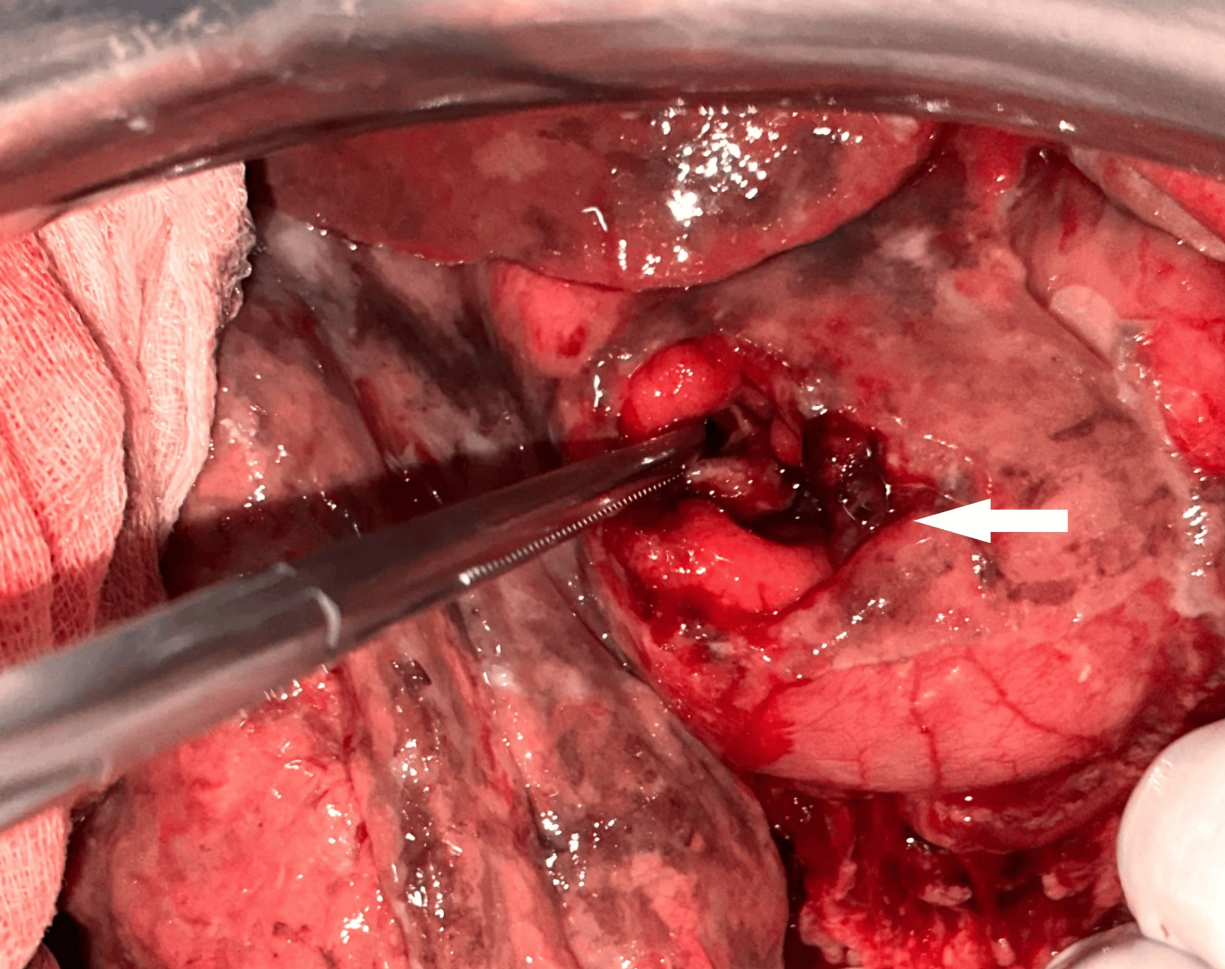
An intraoperative image demonstrating a perforation of the anterior gastric wall

**Figure 3:**
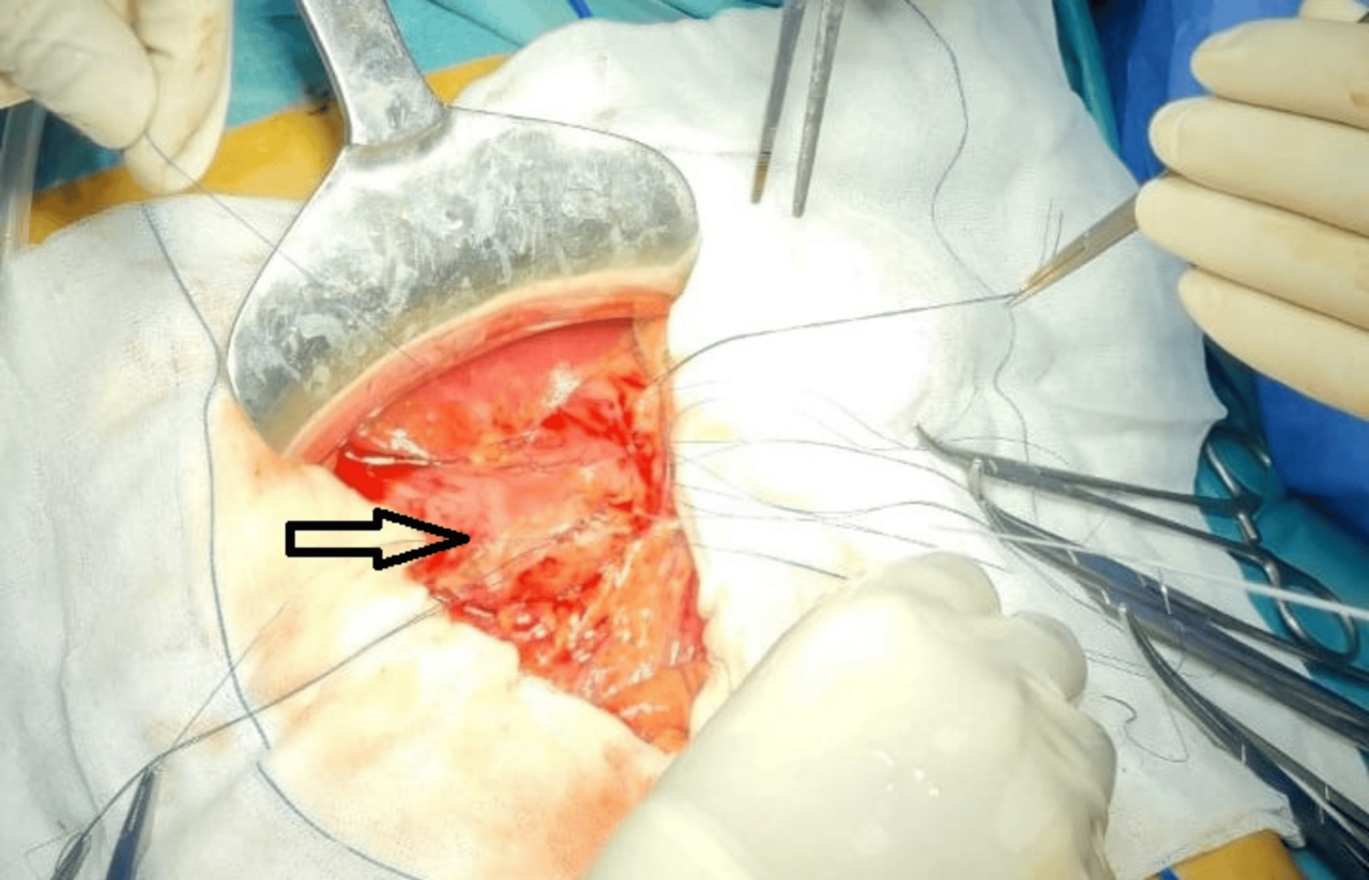
An intraoperative view of the gastric suture performed during surgical repair

Exploratory laparotomy was performed, confirming a large volume of peritoneal effusion with food particles, a 4 cm perforation of the gastric antral wall, and no additional injuries (Figure 2). The perforation was repaired with a two-layer suture after debridement (Figure 3), complemented by omentoplasty, followed by peritoneal lavage and drainage.

The patient’s postoperative care included analgesics, broad-spectrum antibiotics, and parenteral nutrition. A nasogastric tube remained in place for two days to aspirate gastrointestinal secretions. He was discharged after four days with no complications, and the two-week follow-up was unremarkable.

## Discussion

Gastric perforation resulting from blunt abdominal trauma is a rare occurrence, with a prevalence ranging from 0.4% to 1.7% of blunt trauma cases. Isolated gastric perforation is particularly uncommon and is more often associated with splenic injury, followed by thoracic trauma [2,3].

This low prevalence can be attributed to the thicker gastric walls, the protective role of the rib cage, and the relative mobility of the stomach [4]. Gastric perforation typically occurs through one of three mechanisms [5]: rapid deceleration, leading to differential movements between adjacent structures and generating shear forces that can cause tearing of both hollow and solid visceral organs as well as vascular pedicles, particularly at relatively fixed points of attachment; external compression, which leads to a rapid and significant increase in intra-abdominal pressure; and crushing of intra-abdominal contents between the anterior abdominal wall and the spinal column.

In the context of trauma, gastric perforation carries the highest mortality rate among injuries to hollow viscera [6]. Therefore, delays in surgical intervention are directly associated with increased morbidity and mortality [3,4,7].

Gastric perforations can occur at any location within the stomach. They affect the anterior wall in 40% of cases, the greater curvature in 23%, the lesser curvature in 15%, and the posterior wall in 15% [4].

While hemodynamic instability is common upon admission, it has been reported in fewer than 20% of cases [5]. In instances of clear peritonitis, rapid laparotomy is recommended for prompt diagnosis and appropriate management. However, physical examination may be misleading, particularly in patients who are intoxicated or have concomitant injuries such as head trauma or spinal cord injury.

Currently, CT is considered the gold standard for diagnosing cases with uncertain findings in hemodynamically stable patients. Key CT indicators of gastric perforation include unexplained intraperitoneal fluid, pneumoperitoneum, intestinal wall thickening, mesenteric fat stranding, mesenteric hematoma, and extravasation of intestinal contents. Nevertheless, clinical vigilance is essential, as a negative CT scan may fail to identify a gastrointestinal perforation in up to 13% of cases [8].

The surgical approach to gastric injuries depends on the severity, extent, and location of the lesion, as well as the presence of any concomitant injuries. During laparotomy, it is crucial to evaluate for additional gastric lesions. While most gastric injuries can be repaired in two layers with proper debridement, this may not be possible in cases with substantial tissue loss. Such injuries are often associated with other organ or vascular damage, in which case a subtotal or total gastrectomy may be required, depending on the extent and location of the rupture [9].

Sepsis is the most frequent complication, with intra-abdominal abscesses occurring in up to 24% of cases. The incidence of complications is higher in patients with a full stomach, as elevated gastric pH fosters a higher bacterial load, which in turn increases the risk of contamination and subsequent infectious complications [9].

## Conclusions

Isolated gastric perforation is a rare but significant injury that should not be overlooked in cases of blunt abdominal trauma. The prognosis depends on the severity of the injury and the timing of surgical intervention. This case underscores that early recognition and prompt surgical repair can lead to full recovery without complications.
